# Valorization of agri-food crucifer vegetables waste for food, functional food and nutraceuticals applications

**DOI:** 10.1186/s40643-025-00895-4

**Published:** 2025-06-01

**Authors:** Lereen Khaled, Nada M. Ali, Reem Nader, Ninon Rolet, Elizabeth S. Sadek, Mohamed A. Farag, Tamer Shoeib

**Affiliations:** 1 Department of Chemistry, The American University in Cairo, New Cairo, 11835 Egypt; 2 Pharmacognosy Department, College of Pharmacy, Cairo University, Kasr El Aini, Cairo, P.B. 11562 Egypt

**Keywords:** Agri-food waste, Bioactive compounds, Crucifer vegetables, Functional foods, Valorization

## Abstract

Valorization of food byproducts, especially fruits and vegetables, has recently attracted considerable attention, mostly due to their high wastage rates. Exploitation of these byproducts, including the non-edible parts of crucifer vegetables, may provide value-added opportunities in the food, functional food, and nutraceutical industries as well as in non-food applications such as therapeutics, biofuels, and paper pulp production. This review focuses on the state-of-the-art valorization practices of crucifer vegetable agro-food wastes including those of broccoli, cauliflower, cabbage, kale, Brussels sprouts, collards, watercress and radish constituting the main cultivated crucifer vegetables worldwide and suggests potential novel uses through upcycling. A detailed comparative phytochemical composition of crucifer vegetable waste products as potential sources of raw materials in promising applications including the production of food enhancers, and antioxidants is presented. Different extraction techniques combining downstream and white biotechnology processes for the optimum utilization of such agro-food waste are discussed. The valorization of cruciferous vegetables by-products is shown to be economical, sustainable and a viable approach to unlock novel applications across diverse industries. To fully maximize the potential of these underutilized resources and promote an ecological bioeconomy, more research and development into extraction methods and upcycling techniques is needed

**Graphical Abstract:**

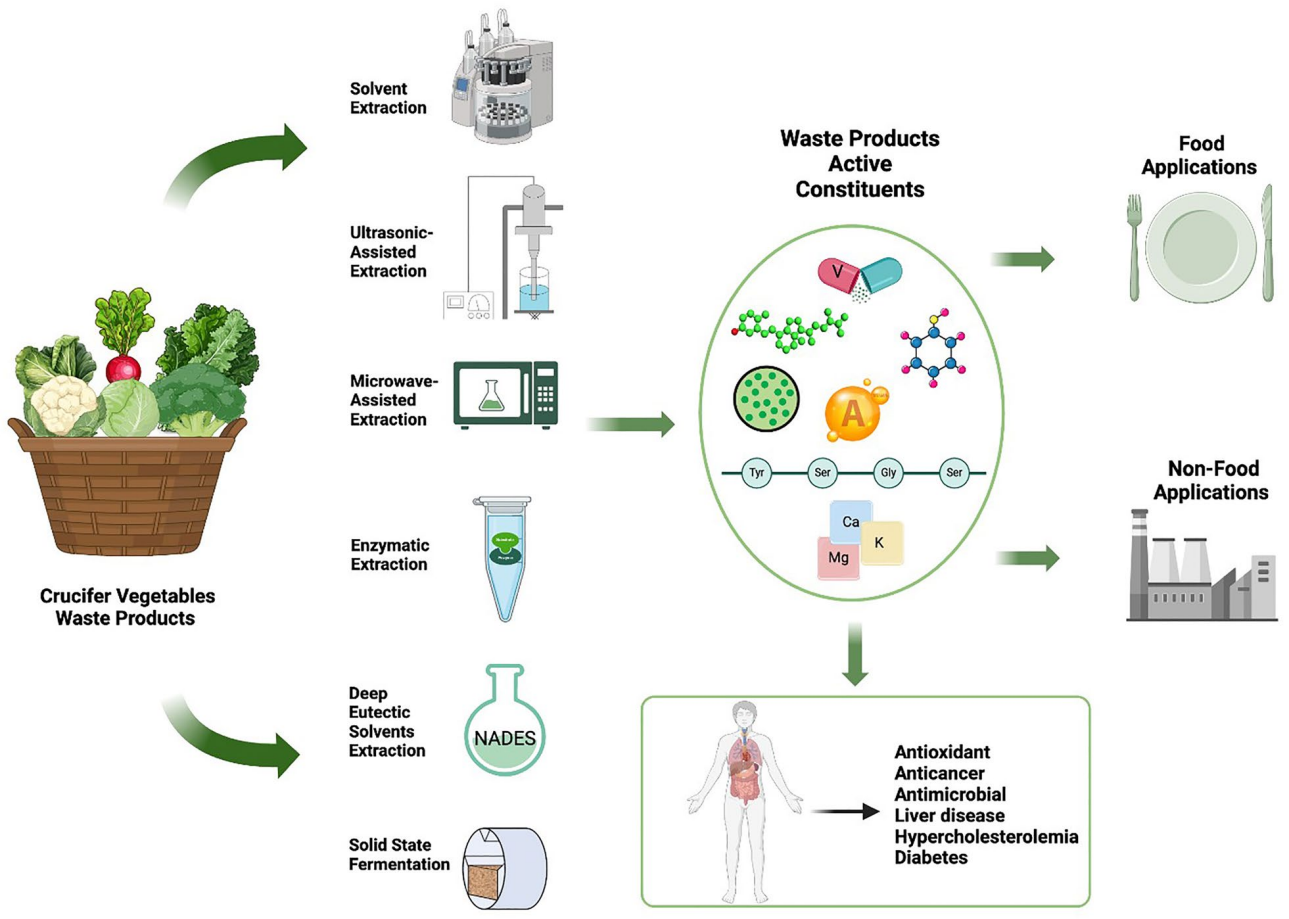

## Introduction

Cultivation, harvesting, processing, storing, marketing, and transporting vegetables produce significant amounts of waste (Mago et al. 2022). In fact, the annual agricultural waste worldwide was reported to reach about 2 billion tons in recent years. In many cases, the non-edible parts of plants, which constitute their major portion, are discarded. For example, broccoli is a plant where only about 30% of the total aboveground biomass, the florets and sprouts, are consumed, whereas the leaves, stalks, and inflorescences, which represent the majority of the plant, are often neglected and regarded as by-products (Zhang et al. 2017). Similarly, the outer leaves of cabbage are usually regarded as food waste even though they contain high nutritional value, as listed in Table 1 (Chaisamlitpol et al. 2014). In fact, it was recently reported that cabbage and cauliflower generate 30 to 50% of their mass as waste by-products (Mago et al. 2022).

**Table 1:** Chemicals found in cruciferous vegetables wastes

Source	Isolated compound	Quantitative amount	References
Broccoli	Phylloquinone Vit K1	2.21 μg/g dw ‡; 24.3 μg/g dw*	(Liu et al. 2018a, b)
	Carotenoids	1095 μg/g dw ‡; 15.6 μg/g dw*	
	Chlorophyll	144 μg/g dw ‡; 4478 μg/g dw*	
Cabbage	Phenolic compounds	19,199.7 μg GAE/g dw	(Chaisamlitpol et al. 2014)
	Glucosinolate	1583.22 mol/100 g dw	(Tanongkankit et al. 2012)
	Vitamin C	5328.5 ± 84.9 μg/g dw	(Nilnakara et al. 2009)
Cauliflower	Carotenoids	5–8 μg/g	(Li et al. 2001)
	Phenolic compounds	4570 μg GAE/g dw*	(Huynh et al., 2014b)
Watercress	Phenolic compounds	1612 μg GAE/g dw^†^	(Zeb 2015)
	Vitamin C	101.5 ± 90 μg/g dw	(Kyriakou et al. 2022b)
Radish	Calcium	7526.4 μg/g	(Gamba et al. 2021)
	Sulforaphene	2074.58 μg/g dw	
	Β-carotene	63 μg/g fw	
Kale	Phenolic compounds	34,320 and 20,870 μg GAE/g rm ‡	(Barrios et al. 2022)
	Glucosinolate	2.83–50.65 μmol/g	(Chang et al. 2019)
	Carotenoids	2.3–339.3 μg/g fw	
	Vitamin C	399.7–1558.2 μg/g fw	
Collard	Glucosinolate	2–4.3 μg/g fw	(Dulović et al., 2023)

dw: dry weight, fw: fresh weight, rm: root dry mass per plant, ‡ stem, *leaf, ^†^root

Improper management of this agricultural waste could lead to deleterious effects. For instance, India generates approximately 141 million tonnes (MT) of crop residue annually, about 92 MT of which is burned due to improper sustainable management strategies. This practice has serious consequences, leading to environmental degradation and poses significant risks to human and animal health (Bhuvaneshwari et al. 2019). Sustainable management of agricultural waste includes its conversion into numerous value-added products such as functional foods, nutraceutical products, as well as biofuels and biodegradable plastics (Singh et al. 2021). The valorization of agro-waste has thus been a growing area of research in the past few years. This paper provides a comprehensive overview of the phytochemical composition of crucifer vegetable wastes generated from broccoli, cabbage, cauliflower, watercress, radish, kale and collards as summarized in Table 1, which were reported to be promising sources of bioactive ingredients including essential vitamins, minerals, different antioxidants, and glucosinolates having several associated health benefits (Ağagündüz et al. 2022; Manchali et al. 2012). Various techniques to extract these valuable bioactive compounds from crucifer vegetable wastes are also discussed and highlighted in Table 2. The review also offers suggestions for creative upcycling applications for the valorization of the extracted bioactive compounds.

**Table 2:** Summary of extraction techniques for cruciferous vegetables waste

Source	Extraction technique	Isolated compound	Temp (°C)	Time	References
Broccoli	S.E. (80:20 ethanol: water)	Phenolics	–	20 min	(Fernández-León et al. 2013)
	S.E. (Acetone/saponification)	Carotenoids/Chlorophyl	–	–	
	S.E. (Ethanol)	Chlorophyll/Tocopherols/Carotenoids	–	–	(Guzman et al. 2012)
	S.E. (Methanol)	Vitamin C/flavonoids/anthocyanins/phenols	–	–	(Porter 2012)
Cauliflower	Enzyme-Assisted	Phenolics/pectin Pectin	40 50	24 h 4 h	(Huynh et al., 2014b) (Zykwinska et al. 2008)
Cabbage	MAE (Ethanol)	Glucosinolates/phenols/sulforaphane	–	5 min	(Chaisamlitpol et al. 2014)
	S.E (CH₂Cl₂ and Na₂SO₄)	Sulforaphane	–	–	(Tanongkankit et al. 2011)
Kale	MAE (alkali)	Phenolics	109	15 min	(Barrios et al. 2022)
Collard	MAE	Glucosinolates	–	–	(Đulović et al. 2023)
Watercress	S.E (Hexane) S.E (methanol)	Phenylethyl isothiocyanate Phenolics/flavonoids	– –	– –	(Kyriakou et al. 2022a, b)
Radish	S.E (80:20 ethanol:water)	Phenolics	–	1 h	(Chihoub et al. 2019)

## Phytochemicals in crucifer wastes

### Phenolic compounds

Phenolic compounds, defined by the presence of at least one aromatic ring and one or more hydroxyl groups, are considered among the most significant of phytochemicals with antioxidant capacity. Phenolic compounds are synthesized in plants as secondary metabolites through the shikimic acid pathway. Phenylalanine ammonia-lyase is the crucial enzyme that facilitates the production of phenolic compounds from the aromatic amino acid phenylalanine (Cartea et al. 2011). They are frequently found conjugated to sugars and organic acids and can be categorized according to the number and arrangement of carbon atoms into flavonoids including flavonols, flavones, anthocyanidins, flavanones, and isoflavones as well as non-flavonoids such as phenolic acids, hydroxycinnamates, and stilbenes (Liu et al. 2018a, b).

### Broccoli

Broccoli byproducts contain the most abundant and diverse spectrum of polyphenols, which consist primarily of flavonoids, mainly flavonols, some anthocyanins, as well as hydroxycinnamic acid (Yan et al. 2023). Flavonols, a subgroup of flavonoids composed primarily of 3-hydroxy flavonoids, are the primary flavonoids found in broccoli byproducts, including quercetin, Kaempferol, and isorhamnetin. They are mainly bound to glucose and frequently acylated with various hydroxycinnamic acids (Cartea et al. 2011). An evaluation of the phenolic profile of the byproducts in three distinct cultivars of broccoli wastes determined that the total concentration of phenolic compounds in broccoli leaves was almost 10 times higher than in the stalks (Vanduchova et al. 2019). A different investigation on fifteen different types of broccoli revealed that, in most cases, the flavonol concentration was greater in the leaves than in the florets. Among these varieties, kaempferol accumulated more in the leaves than quercetin, and in some varieties, the kaempferol concentration was greater in the florets than in the stalks (Duan et al. 2021). A comparison of the nutritional composition of various parts of broccoli, such as the floret, stem, and leaf, showed the total phenolic concentrations in leaves to be 1.6 and 2.9 times higher than in the florets and in the stems, respectively (Liu et al. 2018a). This suggests that there is a considerable amount of genetic diversity in the antioxidant compounds found in broccoli tissues. The most common types of anthocyanins, which are sugar-conjugated derivatives of anthocyanidins, found in plants include pelargonidin, cyanidin, delphinidin, peonidin, petunidin, and malvidin. Cyanidin is the most prevalent in broccoli byproducts, albeit in small amounts. The predominant type of hydroxycinnamic acids found in Brassica byproducts, such as broccoli stems and leaves, are *p*-coumaric, sinapic, and ferulic acids, which are frequently present in conjunction with sugar or other hydroxycinnamic acids (Yan et al. 2023).

### Cabbage

Several phenolic compounds isolated from cabbage waste extracts demonstrated antioxidant, anti-inflammatory, anticancer, and hepatoprotective action. For example, methanolic extracts of cabbage outer leaves were reported to contain 2,3-dihydro-3,5-dihydroxy-6-methyl-4H-pyran-4-one and 2,4-bis(1,1-dimethylethyl)-phenol representing 4.8 and 12.8% respectively of the total volatile compounds. Additionally, flavonols, a subclass of flavonoids polyphenols with an extra ketone group, were found to be the predominant class of phenolic groups in this agro-waste (Liang et al. 2019). Kaempferol glycosides and 14 different quercetins were identified among the flavonol compounds in this methanolic extract (Agati et al. 2016; Kowalski et al. 2021). The levels of flavonoids in the outer leaves agro-waste ranged from 565.2 to 1060.7 mg kg^−1^, which is remarkably higher than in the marketable heads, where it ranged from 64.7 to 146.8 mg kg^−1^ (Kosson et al. 2017). In the case of the outer leaves of Chinese cabbage, different phenolic acids were identified, such as caffeic acid, sinapic acid, *p*-coumaric acid, and ferulic acid, while the main flavonoid that was isolated from it was myricetin (Seong et al. 2016). Pyrolysis oil derived from cabbage waste was found to be rich in phenolic compounds, with concentrations varying significantly according to temperature. The highest phenolic content (55.8%) was achieved at 520 °C, while at 700 °C, the yield dropped sharply to just 32.3% (Zhang et al. 2022). White cabbage residues were also identified as a potential source of phenolic compounds. The average phenolic compounds detected in fresh white cabbage outer leaves were 5.72 and 19.1 mg/g d.w., whereas dried outer leaves at temperatures of 70, 80, and 90 °C showed total phenolic compounds of 7.7, 6.6 − 17.2, and 4.4 mg/g d.w. respectively (Nilnakara et al. 2009; Chaisamlitpol et al. 2014). The hydroethanolic extract of red cabbage was found to be enriched with anthocyanins, which are very well-known anti-aging, antioxidant, and antiradical polyphenol. It was reported to contain 94 mg cyanidin-3 glucoside/L that was stable during 20 weeks of storage, showing only 4% loss over the testing period (Patras 2019). This stability is exceptional relative to other sources of purified anthocyanins such as rice, blueberries, and strawberries.

### Cauliflower

The non-edible parts of cauliflower consisting of outer leaves, stems and pods, have been found to be an excellent source of polyphenols and especially flavonoid glycosides that are mainly composed of quercetin and kaempferol (Francisco et al. 2009). Cauliflower exhibits a remarkable diversity of flavonoid content with upwards of 8,000 unique flavonoid structures, among these, quercetin and kaempferol are the most abundant, being frequently conjugated with glycosides and acyl groups and in particular kaempferol-3 feruloyldiglucoside and kaempferol-3-glycoside appear to be the predominant (Olsen et al. 2010). Other species such as kaempferol-3-O-diglucoside, kaempferol-3-O-diglucoside-7-O-glucoside protocatechuie acid, quercetin, pyrogallol, vanillic acid, and coumaric acid were also identified from cauliflower ago-waste (Huynh et al. 2016). The outer leaves of cauliflower have also been reported to be a promising source of hydroxycinnamic acids such as caffeic, sinapic, p-coumaric and ferulic acids (Lee et al. 2011).

### Watercress, radish, kale, and brussels sprouts

Watercress roots also contain phenolic compounds, including gallic, p-coumaric, caffeic, sinapic acids and their derivatives, hydroxybenzoic acid derivatives, caftaric acid, pro-anthocyanidin trimer, dihydro kaempferol hexoside, kaempferol-3-(caffeoyldiglucoside)−7-rhamnoside, hydroxybenzoic acid, vanillic acid, spincetine glucuronide, quercetin-3,7-diglucoside, quercetin-3-O-rutinoside 7-O-glucoside, and quercetin-3-O-triglucoside (Cuong et al. 2019; Zeb 2015).

Phenolic compounds are also prominent in radish leaves and contribute to their antioxidant properties. Radish leaves contain various phenolic compounds, including epicatechin, coumaric, vanillic, and sinapic acids. Flavonoids, such as quercetin, catechin, and chlorogenic acid derivatives are also reported to be present in radish leaves in high quantities (Goyeneche et al. 2015).

Kale and Chinese kale have both been shown to contain abundant levels of polyphenols, whereas in the Chinese variety, the phenols were shown to be mostly in the leaves and petioles and much less abundant in the roots (Chang et al. 2019).

In Brussels sprout byproducts, 21 bioactive constituents were identified, including 14 flavonoid glycosides such as kaempferol-O-glycosides, kaempferol-O,C-glycosides, and quercetin-O-glycosides, along with ferulic acid, sinapic acid, their glucosides, and aglycones (Gonzales et al. 2015).

### Vitamins

#### Broccoli

One of the primary antioxidants found in broccoli byproducts, especially the leaves, is Vitamin C, which consists of L-ascorbic acid (AA) and its oxidized form, L-dehydroascorbic acid (DHA). AA is an aldono-1,4-lactone of hexonic acid, exhibiting a high propensity for oxidation in aqueous solutions to form monodehydroascorbate, which can undergo in vivo enzymatic reduction back to AA or be spontaneously disproportionate to AA and DHA. Consequently, DHA can be readily transformed into AA within the human body exhibiting three primary biological functions, namely serving as an enzyme cofactor, a scavenger of free radicals, and an electron donor or acceptor. In addition, AA can improve the absorption of iron and calcium within the gastrointestinal tract (Rybarczyk-Plonska et al. 2014).

Broccoli also contains four tocopherols and four tocotrienols, with α- and γ-tocopherols being the primary compounds. According to the USDA nutrient database, the suggested daily intake of vitamin E for adults is 15 mg. A serving of 100 g of fresh broccoli florets, stems, or leaves can supply 0.15, 0.14, or 19.9% of the Recommended Dietary Allowance (RDA), respectively. Research has indicated that Vitamin E is advantageous in combating cardiovascular disease, inflammation, diabetes, prostate cancer, and Alzheimer's. Tocopherol levels varied significantly among different broccoli tissues. Florets, stems, and leaves contained 1.57, 1.97, and 155 μg/g dw of tocopherols, respectively. While α-tocopherol was undetectable in florets, it accounted for 90.4 and 100% of the tocopherols found in the leaves and stems respectively. The leaves showed the highest concentrations of both α- and β-tocopherols (Liu et al. 2018a, b), indicating their potential as an excellent dietary source of vitamin E.

Phylloquinone (vitamin K1), the predominant dietary form of vitamin K, plays crucial roles in blood coagulation, bone health, and prevention of vascular calcification and cardiovascular disease. The USDA recommends daily intakes of 90 μg for adult women and 120 μg for men. Based on 100 g portions of fresh tissues, the leaf and floret tissues can supply 521 and 137% respectively for adult women, and 391 and 102% respectively for adult men of their RDA. Stem tissues on the other hand provide 27 and 20% of the RDA for adult women and men respectively. The floret, stem, and leaf tissues were reported to also have phylloquinone concentrations of 8.84, 2.21, and 24.3 μg/g dw, respectively indicating that broccoli leaf tissues can be a significant source of phylloquinones (Liu et al. 2018a, b).

### Cabbage

Cabbage waste extract was reported to contain different water-soluble as well as lipid soluble vitamins (Liang et al. 2019). For example, white cabbage outer leaves were reported to contain 532.85 ± 8.49 mg/100 g dw of vitamin C which can satisfy the RDA (Nilnakara et al. 2009). In fact, the vitamin C content in the first outer leaf was reported to be 61.3 mg/100 g FW and steadily decline in inner leaves reaching 27.19 mg/100 g FW by the 20 th leaf layer (Zhao et al. 2020). This is in line with another report showing the level of ascorbic acid in the outer leaves of white cabbage to range from 80.02 to 123.3 mg/100 g dw, while the marketable heads and the outer leaves taken together contained 47.06 to 53.86 mg/100 g dw of ascorbic acid (Kosson et al. 2017). These results highlight that the outer leaves of cabbage that are typically agro-waste, contain more vitamin C than the marketed head containing only the inner leaves.

### Watercress, radish and kale

Watercress waste is also a good source of vitamins and minerals, containing vitamins C, B, E, and pro-vitamin A all with well-known potential health benefits (Kyriakou et al. 2022a). Radish leaves are also rich in vitamins especially vitamin C, which is found in significantly higher amounts in radish leaves, compared to its roots (Gamba et al. 2021; Goyeneche et al. 2015).

Chinese kale was reported to contain phenolic compounds, vitamin C, chlorophylls and carotenoids with the typically marketed leaves and petioles containing the highest levels (Chang et al. 2019; Satheesh and Workneh Fanta 2020).

### Glucosinolates

Glucosinolates are one of the major active constituents of the cruciferous plants. They are sulphur-containing glycosides that are considered the main bioactive compounds responsible for the anticancer activity of these plants. All characterized glucosinolates have a common basic structure that includes a d-thioglucose group, a sulfonated oxime group, and an amino side chain (Moreno et al. 2006). They can be classified into three categories according to their amino acid precursors: aliphatic glucosinolates, aromatic glucosinolates, and indolic glucosinolates (Li et al. 2021). These are usually biologically inactive until they are hydrolyzed with the myrosinase enzyme, an endogenous enzyme that is released upon physical damage to the plant, into different active compounds such as isothiocyanates, thiocyanates, nitriles and sulforaphane (Wennberg et al. 2006). Glucosinolates can also be catalyzed in the human gut into different active products (Yan et al. 2023).

### Broccoli

Broccoli byproducts were reported to contain glucosinolates. In fact, the roots were shown to contain nearly 43% of the total glucosinolate content in the plant with glucoraphanin being the predominant form (Li et al. 2021). Glucoraphanin in broccoli is usually hydrolyzed to produce sulforaphane which has two possible optical stereoisomers due to the presence of a stereogenic sulfur atom. While the S-sulforaphane can be synthesized, both The R- and S-sulforaphane enantiomers occur naturally (Janczewski 2022). Interestingly, only one study focused on the separation and quantification of racemic mixtures of sulforaphane derived from broccoli (Okada et al. 2017). In fact, very limited studies have been conducted on the different physiological activities of the enantiomers of sulforaphane (Vanduchova et al. 2019).

Nitriles that are typically not biologically active are also produced through the glucosinolate precursors, this production is enhanced markedly in specific broccoli cultivars due to the existence of epithio-specifier proteins (Vanduchova et al. 2019). Nitrile formation in broccoli floret and leaf tissues was found to be significantly higher in comparison to broccoli stems. This observed variation in nitrile production may potentially be attributed to the tissue-specific expression of myrosinase and/or the levels of myrosinase cofactors which play a regulatory role in its activity. However, it is also possible that distinct myrosinases in different tissues may exhibit varying affinities towards different glucosinolate substrates (Liu et al. 2018a, b).

### Cabbage

While the literature on the bioactive compounds present in the roots of cabbage is very scarce, it is reported that the outer leaves of cabbage contain glucosinolates and sulforaphane in smaller quantities than the inner leaves. The content of total glucosinolates in the first leaf layer was reported to be 3.88 and 2.30 μmol/g dw in comparison to 15.58 and 11.52 μmol/g dw in the 20 th leaf layer for two different cabbage cultivars (Zhao et al. 2020). This is in line with studies reporting the content of glucosinolates to be 8.4, 15.83 and in the range of 10.74 to 16.90 μmol/g dw while the sulforaphane in fresh outer cabbage leaves was reported to be 2.1 μmol/g dw (Chaisamlitpol et al. 2014; Tanongkankit et al. 2012). Glucoraphanin is reported to be the major glucosinolate compound present in the outer leaves of cabbage which is hydrolysed by the myrosinase enzyme into an intermediate that can either rearrange to produce the anticarcinogenic sulforaphane or bind to epithiospecifier protein myrosinase cofactor producing sulforaphane nitrile which has no anticarcinogenic activity (Tanongkankit et al. 2011).

### Cauliflower, watercress, kale, Brussels sprouts and collards

Cauliflower contains several glucosinolates, with sinigrin, progoitrin, and glucobrassicin being the most abundant (Kushad et al. 1999). Glucobrassicin whose concentration is maximized by steaming the cauliflower breaks down with the help of the enzyme myrosinase to form beneficial compounds like indole-3-carbinol, indole-3-acetonitrile, 3,3′-diindolylmethane, and ascorbigen (Aggarwal & Ichikawa 2005).

Watercress roots are rich in glucosinolates, glucotropaeolin, gluconasturtin, glucobrassicin, and 4-methoxyglucobrassicin (Faizy et al. 2021; Kyriakou et al. 2022a). The primary bioactive compounds found in kale, predominantly in their roots, are glucosinolates with 13 types reported in the Chinese variety and gluconapin being the most abundant (Chang et al. 2019).

Several gluconisolates, quinic and a breakdown product of chlorogenic acid have been identified in Brussels sprouts (Gonzales et al. 2015). To the best of our knowledge, only one published report investigated the components of collard byproducts, and it exclusively focused on glucosinolates. In this report 10 different glucosinolates were determined in the non-edible parts of collard with gluconasturtiin and glucoerucin being the main ones at 43 and 26% of the total respectively. Similar to Brussels sprout, collard showed tissue-dependant variation of its glucosinolates profile and concentration between leaves and roots. In the roots, the glucosinolates identified as the most important were gluconasturtiin and glucoerucin. The total glucosinolates levels in collard roots is reported to be more than twice than that found in the edible tissues (Lee et al. 2017).

### Carotenoids

Carotenoids play an important role in various processes related to photosynthesis and the growth of plants, including photomorphogenesis, photoperiodism, and photoprotection. Additionally, they function as precursors in the biosynthesis of abscisic acid and retinol (vitamin A) in addition to their potential health benefits against cancer and cardiovascular and photosensitivity disorders. Carotenoids are considered the second most prevalent natural pigment on the planet and encompass over 750 compounds (Luo et al. 2019).

### Broccoli and cauliflower

The presence of β-Carotene, violaxanthin, neoxanthin, and lutein was identified in various tissues of broccoli where the concentration of total carotenoids in leafs, florets, stems was reported to be 1095, 181 and 15.6 μg/g dw. Lutein was the dominant carotenoid in all tissue types, comprising 47.2, 69.2, and 44.2% of the total carotenoids in the floret, stem, and leafs, respectively. The stem tissues lacked β-carotene and violaxanthin, while the leaf tissues exhibited the highest levels of β-carotene, violaxanthin, neoxanthin, and lutein at 248, 206, 156, and 484 μg/g dw respectively. The concentrations of vitamin A in leaf tissues and floret contribute up to 32.0 and 3.9% of the RDA for adult males and 42.9 and 3.0% of that of adult females respectively (Liu et al. 2018a, b). Thus, the broccoli leaf tissues are a superior source of vitamin A compared to its floret.

Cauliflower is packed with carotenoids such as neoxanthin, violaxanthin, and β-carotene. These three carotenoids contribute over half of the total carotenoid content in cauliflower. An orange cauliflower variety was reported to be particularly rich in β-carotene at 5–8 μg/g fresh weight while containing smaller amounts neoxanthin, violaxanthin, and lutein (Li et al. 2001).

### Chlorophyll

Chlorophyll, responsible for the green color of plants, is a complex compound consisting of a porphyrin ring, a magnesium ion, and a hydrocarbon tail. The porphyrin ring is responsible for the absorption of light, while the magnesium ion functions as an electron acceptor. Chlorophyll exists in various forms (chlorophyll a, b, c, d and e). Chlorophyll a is the predominant type found in plants. Chlorophyll b has a very similar chemical composition possessing a slightly different porphyrin ring (Martins et al. 2023). In Broccoli, chlorophyll a concentration in the florets, stems, and leaves, were reported to be 852, 144, and 4478 μg/g dw respectively while chlorophyll b concentrations were reported to be about 5–7 times lower at 135, 23, and 781 μg/g dw, respectively (Liu et al. 2018a, b). Chlorophyll is also abundant in radish leaves with concentrations higher than in some other green leafy vegetables, such as Swiss chard and butter lettuce (Gamba et al. 2021).

### Essential minerals

Primary elements such as Na, K, Ca, Mg, Cl, and P are necessary for humans in quantities exceeding 50 mg/day. In contrast, trace elements such as Fe, Zn, Cu, Mn, I, F, Se, Cr, Mo, Co, Ni are necessary for concentrations below 50 mg/day. Broccoli, like other members of the Brassicaceae family, contains a high concentration of several essential minerals (Moreno et al. 2006). Among nine minerals extracted from various broccoli tissues, no statistically significant variations were observed for the concentrations of Cu, Mg, or K, the concentration of Fe, Zn, and P were reported to be the highest in floral tissues; leaf tissues had the highest levels of Mn and Ca, and the stem had the highest levels of Na. The concentrations of Ca and Mn in leaves were found to be six times higher and 1.4 times higher, respectively, compared to florets. This observation suggests that the transportation of Ca and Mn to leaves occurs through the transpiration stream in the xylem, as opposed to the phloem, where it is delivered to the florets (White et al., 2003). Calcium and manganese are both immobile in the phloem, and their delivery to developing florets will be restricted compared to other mineral elements like zinc and phosphorus. The limited Na concentrations in leaves and especially florets point to the existence of effective Na exclusion systems in broccoli, which may contribute to its healthy mineral-element profile (Liu et al. 2018a, b). Essential minerals such as potassium, magnesium, copper and calcium were reported to be present in non-edible raddish leaves with calcium being the most abundant at 7.53 mg/g dw (Gamba et al. 2021).

### Amino acids and bioactive peptides

Cauliflower waste, including its leaves, contains significant amounts of peptide hydrolysates, which become activated upon the release of the larger proteins that encase them. Such peptide hydrolysates include angiotensin-converting enzyme inhibitory peptides (Montone et al. 2018), which can help regulate blood pressure, and calcium-binding phosphopeptides which may play a role in bone health (Volden et al. 2009). Broccoli stems and leaves are rich in additional functional nutrients and components, including crude proteins, which comprise an average of 23% of the total bioactive compounds. Plant proteins typically demonstrate limited bioactivity due to their inadequate solubility in aqueous solutions. Recently enzymatic hydrolysis of plant proteins using various proteases has been employed to enhance the solubility and functionality of these proteins (Hua et al. 2018). This approach has yielded functional peptides that exhibit significant nutritional value with potential human health benefits such as antioxidant and hypolipidemic effects. Therefore, the potential for broccoli peptides in developing functional foods or nutraceutical products for direct human consumption is recognized (Chen et al. 2020). Radish also is rich in proteins with the leaves having higher protein, ash, and crude fiber content compared to the edible roots (Goyeneche et al. 2015).

## Extraction techniques for crucifer phytochemicals.

Analyzing medicinal plants relies heavily on extraction, the initial step where valuable chemical components are separated from the plant material. This allows the isolation and characterization of the active ingredients. Before extraction, the plant material undergoes washing to remove dirt and other impurities, drying to preserve the plant, and grinding to create a uniform sample for better solvent extraction. These steps are typically followed by the extraction, which separates the desired parts of the plant from inactive materials while ensuring minimal damage to the potential active components (Aggarwal & Ichikawa 2005; Kushad et al. 1999). The choice of extraction method depends on various factors like the plant itself, the solvent used, temperature, and even the intended use of the final product. There is a wide range of extraction techniques available including maceration, percolation, and ultrasound-assisted methods. Following extraction, separation techniques like chromatography come into play to further isolate and purify the desired compounds of potential benefit (Azwanida 2015; Doughari 2012; Ingle et al. 2017; Pandey & Tripathi 2014; Sasidharan et al. 2010).

### Solvent extraction

Solvent extraction employs solvents such as ethanol or water to dissolve and separate the target components from the source material by using the selective dissolution of the desired compounds in these solvents. Solvent extraction is used to extract different scents, colors, and bioactive substances from a variety of food and botanical sources. This method is widely used with broccoli (Gasser & Abdel Rahman 2021). Toluene and methanol, followed by acetylation, were successfully used to extract 13 fatty acid methyl esters and sterols from broccoli roots (Campas-Baypoli et al. 2009). Lutein and beta-carotene were also extracted from broccoli heads using acetone or tetrahydrofuran (Fernández-León et al. 2013; Pellegrini et al. 2010). Phenolic and flavonoid compounds were also reported to be extracted from broccoli using methanol and water mixtures, however, the isolation of the latter compounds required an additional step of mixing the broccoli extract with sodium nitrite, aluminum chloride, and sodium hydroxide (Oh & Rajashekar 2009; Aires et al. 2011; Naguib et al. 2012). Vitamin C was reported to be extracted from broccoli employing meta-phosphoric acid by the titrimetric method, or a solvent mixture of citric acid, EDTA, NaF, methanol, and water (López-Berenguer et al. 2009). Several solvent systems including mixtures of methanol and water, methanol and hydrochloric acid, hydrochloric acid with potassium chloride and ammonium or sodium acetate, as well as methylene chloride were all employed in order to extract total flavone, flavonoid and anthocyanins from broccoli (Haina et al. 2010; Martínez-Hernández et al. 2011; Porter 2012) while solvent extraction typically using methanol is also one of the most used techniques for extracting cabbage active constituents (Zhang et al. 2022).

### Supercritical fluid extraction (SFE)

Supercritical fluid extraction is a highly effective method for isolating heat-sensitive bioactive compounds such as lipids, due to its reliance on the solvating power of gases above their critical point. When a substance surpasses its critical temperature and pressure, it transforms into a supercritical fluid possessing both gas and liquid properties. SFE, particularly utilizing supercritical CO_2_ at moderate temperatures is ideal for extracting heat-sensitive substances and is more environment friendly compared to conventional solvent-solid extraction methods (Mandal et al. 2015). In a recent study, SFE employing a mixture of CO_2_ and 15% methanol was utilized to extract 22 fatty acids from broccoli leaves (Arnáiz et al. 2011).

### Microwave-assisted extraction (MAE)

Microwave-assisted extraction utilizes microwave energy to disrupt cell structures and heat solvents, enhancing the transfer of analytes from the sample matrix into the extraction solvent (Eskilsson & Björklund 2000; Zhang et al. 2022). This typically achieves accelerated processing and high extraction yields possibly through a synergistic interplay of heat and mass transport phenomena (Chemat et al. 2009). In a comparative study of extraction techniques, MAE was reported to be one of the most efficient extraction methods for sulforaphane from cabbage and gallic acid from cauliflower (Chaisamlitpol et al. 2014; Baiano et al. 2014). However, like other assisted extractions, MAE can involve significant costs and thus while MAE might be very advantageous in small scale applications, its use on a larger scale could be restrictive (Zhang et al. 2022). It is noteworthy that the utilization of drying processes such as hot hair drying and low-pressure superheated steam drying post MAE extraction were shown to typically significantly reduce the final yield of thermolabile bioactive compounds (Chaisamlitpol et al. 2014).

In a study employing MAE to extract bioactive materials from Kale stems the impact of temperature, time, and NaOH concentration were investigated and showed the third parameter to be very significant to the final extract yield reaching a maximum of 22.6% by d.w. of which a total sugar content of 14 g L^−1^ was obtained. This study validates the potential of MAE from kale stems utilizes a water-based solvent under mild conditions as a promising process in a bio-refinery setting (Barrios et al. 2022). MAE was also successfully employed to isolate glucosinolate degradation products from collard yielding 2.0 to 4.3 μg/g FW of volatiles of three aliphatic classes (Đulović et al. 2023).

### Ultrasonic-assisted extraction

Solvent extraction is often supplemented by assisted extraction techniques such as ultrasound sonification to obtain better yields and decrease the extraction time (Olsen et al. 2009; Amofa-Diatuo et al. 2017; Xu et al. 2017; Park et al. 2023). For example, the extraction of glucosinolates from kimchi cabbage was maximized by including an ultrasound sonification step (Park et al. 2023). Ultrasonic-assisted extraction has also been reported to efficiently extract proteins from leaf cauliflower waste achieving 53% recovery (Xu et al. 2017). However, increased sonication amplitudes were reported to suppress the recovery of isothiocyanates from cauliflower by-products (Amofa-Diatuo et al. 2017).

### Enzyme-assisted extraction

Enzyme-assisted extraction is a potential alternative to the use of organic solvents for extraction where different enzymes can be used as catalysts to degrade matrices cell walls for extracting a variety of biologically active compounds from natural sources. Several factors should be considered when selecting the enzyme such as its specificity, activity, botanical origin, working pH, required ratio with the substrate, operational temperature and reaction time required (Streimikyte et al. 2022). Treatment of cauliflower byproducts with two different polysaccharide-degrading enzymes being Viscozyme L and Rapidase were reported to increase the yield of released phenolic compounds to 8.5 and 6.0 mg/g d.w respectively from 4.6 mg/g d.w without enzyme treatment (Huynh et al., 2014a). Enzymatic extraction was also successfully employed in extracting pectin and pectic oligosaccharides from cauliflower florets and leaves (Zykwinska et al. 2008).

### Alkaline extraction

Alkaline conditions are often employed in extractions of different active constituents from agricultural waste. Alkaline environments degrade lignin by partially dissolving hemicellulose to facilitate the extraction process thereby increasing extract yield. This technique was reported successful for the extraction of polyphenols and proteins from cauliflower waste as well as proteins from kale byproducts (Majumdar et al. 2021; Sedlar et al. 2021; Barrios et al. 2022). Alkaline extraction works by saponifying ester linkages and disrupting cell wall polymers, which enhances the release of intracellular compounds such as phenolics and proteins. This process is especially effective for lignocellulosic biomass, making it well-suited for cruciferous vegetable residues. However, this extraction methodology entails lengthy extraction times if unassisted with complimentary techniques such as ultrasonication or MAE (Barrios et al. 2022). It is also important to mention that increasing the pH of the extraction media should be carefully controlled to avoid degradation of the target analytes.

### Solid state fermentation

Solid-state fermentation was reported to be a promising extraction technique for phenolic content from cauliflower where six different fungal strains (*A. niger, A. oryzae, A. sojae, R. oryzae, R. azygosporus and P. chrysoporium*) were employed separately in the fermentation to release and bio-convert the extracted phenolic compounds. The highest yield was obtained using *A. sojae*, which resulted in a rutin content of 3.2 mg/g F.W. after 1 day of fermentation compared to 1.1 mg mg/g F.W. after the same duration in the unfermented sample (Huynh et al. 2016).

Recent findings further support the use of solid-state fermentation for improving the nutritional and functional value of agri-food residues. This approach has been shown to significantly enhance antioxidant activity and total phenolic content by promoting enzymatic transformations particularly through β-glucosidase and lignocellulose degrading enzymes, which facilitate the release of bound phenolic compounds (Sarıtaş et al. 2024). It was shown that fungal strains adopted in conjunction with this approach can have strain dependent effects on bioactive compound release and antioxidant capacity, with some fermentations yielding more than ninefold increases in phenolic content and radical scavenging activity compared to unfermented controls (Li et al. 2023).

### Natural deep eutectic solvents extraction

Natural deep eutectic solvents (NADES) are plant-derived liquids primarily consisting of amino acids, sugars, sugar alcohols, and organic acids. These solvents serve as eco-friendly alternatives to conventional organic solvents in liquid extractions.

NADES exhibit valuable properties such as non-volatility, biodegradability, and high solubilizing capacity, making them ideal for natural product extraction. They are formed by combining two or more natural compounds, which interact through hydrogen bonding to create a eutectic mixture. This mixture exhibits a reduced melting point, freezing point, and vapor pressure compared to the individual components. This unique characteristic allows NADES to remain liquid at or near room temperature, reducing the need for volatile organic solvents (Liu et al. 2018a, b).

The physicochemical properties of NADES, including viscosity, polarity, and density, can be modified by adjusting their molecular composition, providing a flexible foundation for various extraction purposes. Furthermore, their inherent biocompatibility and low toxicity make NADES particularly attractive for food, pharmaceutical, and cosmetic industries, aligning with the growing demand for sustainable and bio-based solutions (Liu et al. 2018a, b).

Different NADESs were reported to be successful for extracting polyphenols, β-carotene, lutein, and chlorophylls from kale leaves waste. Hydrophilic NADESs were synthesized using glycerol combined with betaine, sorbitol, xylose, glucose, fructose, and urea, while hydrophobic NADESs were produced using mixtures of terpenes such as DL-menthol, thymol, and fenchyl alcohol. Glycerol based hydrophilic NADESs were shown to produce extracts rich in polyphenols, while terpene based hydrophobic NADESs selectively recovered lipophilic carotenoids and chlorophyll (Lee et al. 2023).

## Potential health benefits and safety concerns of cruciferous vegetable byproducts

### Therapeutic potential against metabolic and inflammatory stress of liver and kidney diseases

*In-vitro* testing of broccoli leaf, stalk, and inflorescence extracts on human hepatoma HepG2 showed dose-dependent toxicity, with a maximal non-toxic concentration of leaves, stalks, and inflorescences estimated at 10 µg/mL. The three extracts were proven effective in decreasing lipid droplet accumulation in the HepG2 cells at supraphysiological fatty acid concentrations. The leaves extract also successfully reduced the accumulation of reactive oxygen species and the associated oxidative stress. These results align with the chemical analysis results of the three extracts, which confirmed that the leaf extracts containing the highest phenolic content, thus possessing the highest antioxidant capacity (Castelão-Baptista et al. 2023).

The treatment of gentamicin-induced liver and kidney injuries in rats with cauliflower waste extracts containing vanillic, p-coumaric and ferulic acids as well as quercetin was shown to reduce lipid peroxidation, improve glutathione levels in both the liver and kidneys and downregulate the IL-1β and NF-κB markers (Khalil et al. 2022). These results suggest that this eco-friendly approach can be used to develop functional food candidates with high antioxidant capacities.

### Antioxidant activity free radical scavenging and cellular protection

Broccoli leaves extracts have been shown to act as antioxidant agents in several *in-vitro* studies (Duan et al. 2021; Le et al. 2020; Sedlar et al. 2021; Yuan et al. 2010; Zhang et al. 2017). In a recent study extracts from 15 diverse broccoli samples were shown to possess significant antioxidant capacity by means of two approaches the 2,2-diphenyl-1-picryl-hydrazyl-hydrate assay and the ferric reducing antioxidant power test. This was attributed to the abundance of kaempferol and quercetin in the extracts (Duan et al. 2021). In another *in-vitro* study simulated digestion was performed on broccoli leaves to determine the antioxidant activity of its extracted proteins. The results confirmed that proteins extracted show potent antioxidant activity with radical scavenging capacity of 72.9% and an IC50 value of 0.35 mg/mL. It was postulated that post *in-vitro* digestion, the broccoli leaves proteins were broken down into small peptides which possessed greater antioxidant potential than their precursors (Sedlar et al. 2021). While extracts from the edible portions of broccoli do exhibit some antioxidant activity, those derived from the non-edible leaves and seeds were shown to possess the strongest antioxidant activity and radical scavenging ability respectively among the three extracts, clearly displaying the valorization potential of such agro-waste (Le et al. 2020; Zhang et al. 2017).

Extracts from tronchuda cabbage waste was shown to exhibits antioxidant activity mostly attributed to its phenolic and organic acid content. Phenolic compounds such as flavonoids and organic acids scavenge free radicals, reducing oxidative stress and protecting cells from damage (Vrchovská et al. 2006). The functional properties of peptides recovered from cauliflower leaves waste were tested by highly predictive bioassays and in silico analysis as potential nutraceuticals. It was suggested that at least two fractions of these extracts possessed significant antioxidant and anti-inflammatory effects in the vasculature, due to inhibition of intracellular xanthine oxidase activity and modulation of superoxide dismutase-1 and vascular cell adhesion molecule-1 expressions. In silico molecular docking analysis identified four different peptides as the most probable bioactive candidates exerting protective effects against endothelial dysfunction and one peptide that synergistically inhibiting xanthine oxidase activity (Caliceti et al. 2019).

Extracts from non-edible waste of watercress were reported to have antioxidant properties. This was linked to the presence of phenolic, flavonoid and ascorbic acid content (Kyriakou et al. 2022b). Black radish peel extracts were reported to be rich in polyphenolic content and thus had significant antioxidant activity which may be useful in nutraceutical formulations (Yücetepe et al. 2021). In fact, extracts from the stems and leaves of radishes were reported to possess significant metal chelation activity, offer protection against DNA damage caused by H_2_O_2_ and to delay the peroxidation of linoleic acid and thus inhibited this peroxidation in a way comparable to antioxidants such as quercetin and BHT (Beevi et al. 2010a, b).

### Anticancer activity

Recent studies indicate that non-edible parts of broccoli may harbor compounds with stronger in vitro anticancer effects compared to the edible portions (Doughari 2012). In vitro experiments tested extracts from broccoli leaves, seeds, and florets against three cancer cell lines: human lung carcinoma (A549), colorectal adenocarcinoma (Caco-2), and hepatocellular carcinoma (HepG2). Seed extracts demonstrated the highest activity, with LC50 values of 0.134, 0.209, and 0.238 mg/mL against these cell lines (Le et al. 2020). It was also demonstrated that the three extracts activated apoptosis in Caco-2 cells, which displayed a reduction in mitochondrial membrane potential and an increase in the subG1 population (Le et al. 2020). The tested seed extracts demonstrated substantially lower cytotoxicity than doxorubicin, a compound known to exhibit potent cytotoxicity with LC50 values in the micromolar range (Osman et al. 2012). When compared to other natural compounds, these extracts showed weaker activity than highly potent garlic-derived allicin, which displays remarkably low LC50 values (Ravindra et al. 2023), but were more effective than certain plant-based extracts like turmeric (LC50 > 2 mg/mL in HepG2 cells; Aggarwal et al. 2013). This comparative analysis indicates that broccoli extracts possess intermediate anticancer properties, likely attributable to bioactive components such as sulforaphane.

Extracts from tronchuda cabbage were reported to be rich in glucosinolates and derived products such as isothiocyanates and indoles with the potential to inhibit tumor growth, induce apoptosis in cancer cells, and modulate detoxification enzymes, thereby exerting anticancer effects (Ferreres et al. 2006). Watercress waste extracts induced an apoptotic response in human malignant melanoma cells and is recognized as active against prostate, cervical, liver and breast cancers most likely due to high gluconasturtiine content (Kyriakou et al. 2022a).

Kale is considered a potent immune booster increasing the production of immunoglobulins that may help in cancer prevention (Clarke et al. 2011). It was reported that consuming kale once per week leads to 17% lower risk of developing oral, colorectal, and breast cancers, as well as a 28% and 32% lower risk of developing esophageal and kidney cancers, respectively. This might be due to kale's abundance of glucosinolates, which convert into cancer-fighting isothiocyanates in the body. These complex substances are powerful inducers of cancer-destructive enzymes as well as carcinogenesis inhibitors. Sulforaphane, a water-soluble component in kale, is suspected to contribute significantly to this impressive array of health benefits (Bosetti et al. 2012).

### Antimicrobial effects

Not only were broccoli byproducts proven to be potent antioxidants and anticancer agents, but they also showed strong antibacterial activity. Leaf, seed and florets extract of broccoli were reported to be active against both gram-negative bacteria such as *E. coli and S. typhimurium* and gram-positive bacteria such as *S. aureus* and *B. subtilis.* The leaf extract was the most effective of all the studied extracts, as evidenced by the lowest minimum inhibitory concentration (MIC) of 0.78–1.56 mg/mL (Le et al. 2020). Broccoli seeds extract showed potential as a natural food preservative owing to its antifungal properties demonstrated through *in-vitro* study against *F. culmorum* and *P. expansum* fungal strains. It effectively prevented the germination of their spores and showed MIC of 2.31 μM against both strains. This antifungal activity was linked to an isolated napin-like protein which demonstrated heat stability and non-cytotoxicity towards mammalian cells that promoted it as a promising antifungal food decontaminant (Thery et al. 2020). Cabbage leaves waste extracts contain glucosinolates and their derivatives, which exhibit antifungal activity against Candida strains, responsible for mucosal infections, as well as antibacterial activity against gram-positive bacteria, including S. aureus. These antimicrobial properties highlight the potential for such agro-waste in natural remedy formulations for treating such infections (Arrais et al. 2022).

The antimicrobial effects of cauliflower by-product extracts were examined on worms through an in-vivo model. The study demonstrated the protective effects of the extract on aging in uninfected worms, likely due to the antioxidant properties of the compounds found in the extract, and significant reduction in Salmonella cells effects in worms infected with the pathogen (Ibáñez-Peinado et al. 2020). Significant antifungal activities against the *A. flavus, A. niger, A. clavatus, and F. solani* species as well as broad antibacterial action were also reported for the seeds and aerial extracts of white radishes, largely attributable to raphanin (Duy et al. 2019).

### Cholesterol-lowering and antidiabetic potential

Cabbage waste extracts contain compounds, such as dietary fiber and polyphenols, that demonstrate a high bile acid binding capacity. These extracts effectively bind to bile acids, preventing their reabsorption and promoting their excretion from the body. This mechanism helps reduce cholesterol levels by reducing the availability of bile acids for fat absorption, ultimately promoting hypocholesterolemic effects and potentially contributing to cardiovascular health (Liang et al. 2019).

Polysaccharides extracted from broccoli by-products were reported to be promising antidiabetic agents by demonstrating their ability to control postprandial blood levels. Their mechanism of action includes inhibiting α-glucosidase and α-amylase enzymes that are mainly responsible for the hydrolysis of carbohydrates in the GIT and increasing postprandial blood level (Zhang et al. 2017; Zafar et al. 2021). The α-glucosidase inhibitory effect of three different fractions of the polysaccharide extract from broccoli byproducts were significant with reported IC50 values of 2.595, 1.733, and 0.991 mg/mL, respectively. (Zhang et al. 2017) These numbers indicate low to moderate potency compared to some natural and pharmaceutical inhibitors with the most effective fraction (IC50 0.991 mg/mL) being less potent than fenugreek seed extract (Srinivasa & Naidu 2021) or the clinical drug Acarbose (Asadi et al. 2024), both having IC50 values in the micromolar range.

Cabbage waste extracts contain bioactive compounds, such as flavonoids and phenolic acids, that retard glucose dialysis potentially leading to hypoglycemic effects. By slowing down glucose absorption and diffusion, these extracts have the potential to help regulate blood sugar levels, making them potentially beneficial for managing conditions such as diabetes (Liang et al. 2019).

### Potential safety concerns of crucifer waste byproducts

Byproducts of cauliflower are a source of compounds that provide numerous health benefits. The extraction processes employed on agro-waste may contribute to the production of toxic species. For example, the anaerobic co-digestion of mixed cabbage and cauliflower agricultural waste results in elevated methane and nitrogen levels in the reactors which may contribute to the production of inhibitory compounds into the mixing liquor of the reactors. While nitrogen is a crucial nutrient for the diverse groups of microorganisms found in anaerobic digesters, an excessive amount of free ammonia can partially or completely hinder the digestion process, resulting in the disruption of biogas production (Beniche et al. 2021).

Extracts of kale and collards are rich in several minerals, including iron and potassium, however, they may also contain goitrogens, which are natural compounds that can interfere with thyroid function. This may be a concern particularly for individuals with existing thyroid dysfunction or iodine deficiency (Felker et al. 2016). The high oxalate content in kale may bind with calcium, potentially increasing the risk of kidney stone formation in susceptible individuals (Satheesh and Workneh Fanta 2020).

## Crucifer waste in food and functional food applications

### Broccoli

The feasibility of utilizing broccoli byproducts to produce commercial instant functional food soup mixes, other beverages such as tea and soft drinks, sauces such as béchamel as well as solid foods such as crackers, sponge cakes and gluten-free bread were reported. The soup mix was shown to be rich in beneficial constituents such as phenolic compounds and sulforaphane produced via the transformation of glucoraphanin (Alvarez-Jubete et al. 2014; Shinali et al. 2024). Powdered broccoli byproducts were also reported to enhance the nutritional profile of flatbread by replacing 5 to 7.5% of the usual flour content (Saleh 2022). Enriched flour has also been created from cauliflower byproducts which can be used in many baked foods such as bread and pizzas (Nartea et al. 2023). Broccoli byproducts have demonstrated various valuable applications, including the production of food preservatives (Shinali et al. 2024), pectin for use as an emulsifier or thickening agent (Petkowicz & Williams 2020; Shinali et al. 2024), and as part of a carbohydrate-protein matrix for the controlled release of bioactive compounds from tuna oil (Shi et al. 2020).

### Cabbage

The production of dehydrated vegetables and powdered vegetable by-products is accompanied by an enormous amount of wastewater output. The food processing industry generates daily around 700 m^3^ of wastewater containing soluble solids, sugar, proteins, and minerals such as calcium, magnesium, iron, zinc, copper, and manganese (Liu et al. 2023). The valorization of this wastewater includes a mixed fermentation process to produce a fermented mash, which is then further fermented and refined to produce vinegar. Under ideal fermentation conditions, the vinegar produced was reported to contain total acid and ester concentrations that comply with those of the commercial product. In fact, the vinegar produced from dehydrated cabbage wastewater was reported to have higher ester content, better taste, and quality (Liu et al. 2023). Cabbage waste has also been utilized as a medium for cultivating microbial biomass and as a source for producing soluble dietary fiber by enzymatic treatment. The latter could bind to bile acids and delay the process of glucose dialysis and thus have the potential for being transformed into nutraceutical products that can promote health (Liang et al. 2019). L-arginine extracted from cabbage waste was converted into L-ornithine which has health benefits and is found in certain types of kimchi, using *P. pentosaceus* bacteria with pH adjustment with eggshells (Kurata et al. 2022). It has also been demonstrated that the outer leaves of cabbage produce nanocellulose, more precisely nanofibrillated cellulose, which is distinguished by its long, entangled nanofibers with both amorphous and crystalline regions. This type of cellulose has special qualities like a high aspect ratio, a large specific area, noticeable strength, and stiffness that are used in a variety of industries, including food, paint, cosmetics, and pharmaceuticals. (Khukutapan et al. 2018).

### Cauliflower and watercress

Cauliflower waste rich in many bioactive compounds such as carotenoids and glucobrassicin, can be used as source of dietary fiber, a beneficial source of proteins and in many baked foods such as breads and pizzas (Nartea et al. 2023; Stojceska et al. 2008).

Watercress waste also presents a valuable resource for both flavoring and nutrition. While the naturally occurring isothiocyanates in watercress waste can have a bitter or sharp taste, watercress powder, used in small amounts, could be used as a natural flavoring agent for various food and beverage products. Additionally, watercress powder could be used to fortify beverages with its nutrients. Watercress waste powder is a source of concentrated phenethyl isothiocyanate and can be encapsulated as a dietary supplement. This could be particularly attractive for individuals who dislike the taste of fresh watercress or find it difficult to incorporate regularly into their diet (Palliyaguru et al. 2018; Coscueta et al. 2020).

### Radish, collard and kale

Red radish brine, a byproduct of radish pickling contains anthocyanins, which are natural antioxidant pigments whose concentrations increase during lactic acid fermentation.The extraction of these anthocyanins make radish brine a potential source of natural food coloring and antioxidant (Giusti & Wrolstad 2003; Matsufuji et al. 2007). Radish waste powder also acts as a natural nitrite source, potentially replacing the chemical additives used in preserving meats. This could offer a more natural alternative while maintaining the functionalities of preventing harmful bacteria, enhancing color and flavor, and slowing down fat spoilage in meats (Honikel 2008; Pegg & Honikel 2014).

To the best of our knowledge, reports on functional food applications of collard and kale wastes are very limited. Collard wastes are a rich source of vitamins, minerals, and glucosinolates. Extracting different bioactive compounds from kale stems and leaves and utilizing them to fortify various food products was reported (Berndtsson 2019). For example, kale waste was reported to be rich in dietary fibers which were proven to have health beneficial effects such as maintaining digestive health, regulating blood sugar levels, and promoting satiety. This can be particularly useful in enhancing the nutritional profile of different foods such as fiber-rich bread, snacks, or cereal bars. The main challenge to the commercial utilization of kale waste is its undesirable taste and texture which restricts its application (Berndtsson 2019).

## Conclusion and future prospects

Crucifer vegetables are widely recognized for their significant nutritional value and ability to promote health. Their health benefits are attributed to their high levels of bioactive phytochemicals. Several studies have predominantly focused on the edible parts of these vegetables. However, there is a pressing need to explore and utilize the byproduct or waste portions including those of leaves, stems, and roots.

In this review article, we presente the substantial nutritional value and health benefits of byproducts derived from crucifer vegetables such as cauliflower, broccoli, cabbage, collard, kale, watercress, and radish. These byproducts contain bioactive compounds such as glucosinolates, flavonoids, anthocyanins, and carotenoids, which are known for their health-promoting and therapeutic effects, including antioxidant, antimicrobial, and anticancer properties. These potential benefits, coupled with their abundance and sustainability, make these agro-wastes attractive for innovative product development. Future research should focus on the toxicity, potential pesticide residues, allergenicity, bioavailability, and metabolism of the active constituents of these byproducts to ensure their safety and their suitability for functional products. It is clear that the potential of crucifer byproducts extends far beyond their current applications. Some potential future prospects for these byproducts include their use as targeted functional ingredients in personalized nutrition, where advances in genomics and nutrigenomics may enable the development of personalized food products tailored to individual nutritional needs. Technologies such as 3D printing could be used to create innovative food products incorporating crucifer byproducts in unique and appealing ways.

The integration of crucifer byproducts into circular economic models can contribute to a more sustainable food system by reducing waste and promoting resource efficiency all while addressing global food security challenges and potentially leading to innovative nutraceutical products for the betterment of human health.

## Data Availability

All data used in this manuscript are publicly available.
